# Fabrication of Apparatus Specialized for Measuring the Elasticity of Perioral Tissues

**DOI:** 10.3390/ma17153654

**Published:** 2024-07-24

**Authors:** Ryo Takemoto, Junya Kobayashi, Yuko Oomori, Kojiro Takahashi, Isao Saito, Mika Kawai, Tetsu Mitsumata

**Affiliations:** 1 Division of Orthodontics, Faculty of Dentistry & Graduate School of Medical and Dental Sciences, Niigata University, Niigata 951-8514, Japan; 2 Graduate School of Science and Technology, Niigata University, Niigata 950-2181, Japan; 3 Department of Orthodontics, Niigata University Medical & Dental Hospital, Niigata 951-8520, Japan

**Keywords:** elasticity, perioral tissue, mechanical property, human skin, Young’s modulus

## Abstract

On the human face, the lips are one of the most important anatomical elements, both morphologically and functionally. Morphologically, they have a significant impact on aesthetics, and abnormal lip morphology causes sociopsychological problems. Functionally, they play a crucial role in breathing, articulation, feeding, and swallowing. An apparatus that can accurately and easily measure the elastic modulus of perioral tissues in clinical tests was developed, and its measurement sensitivity was evaluated. The apparatus is basically a uniaxial compression apparatus consisting of a force sensor and a displacement sensor. The displacement sensor works by enhancing the restoring force due to the deformation of soft materials. Using the apparatus, the force and the displacement were measured for polyurethane elastomers with various levels of softness, which are a model material of human tissues. The stress measured by the developed apparatus increased in proportion to Young’s modulus, and was measured by the compression apparatus at the whole region of Young’s modulus, indicating that the relation can be used for calibration. Clinical tests using the developed apparatus revealed that Young’s moduli for upper lip, left cheek, and right cheek were evaluated to be 45, 4.0, and 9.9 kPa, respectively. In this paper, the advantages of this apparatus and the interpretation of the data obtained are discussed from the perspective of orthodontics.

## 1. Introduction

On the human face, the lips are one of the most important anatomical elements morphologically and functionally [1]. Morphologically, they have a significant impact on aesthetics [2], and abnormal lip morphology causes sociopsychological problems [3]. Functionally, they play a crucial role in breathing, articulation, feeding, and swallowing. Although it is desirable for the lips to be able to close without an excessive tension in the perioral muscles, various factors can cause the upper and lower lips to be in a constant state of separation, which is called lip incompetence [4]. Incompetent lip has been reported to be associated with oral breathing, tonsillar hypertrophy, allergic rhinitis, maxillofacial morphology, malocclusion, and laxity of perioral muscles, and causes various problems such as gingivitis and exacerbation of periodontal disease due to a dry mouth. Malocclusions that yield lip incompetence include maxillary protrusion, mandibular protrusion, and bimaxillary protrusion. These malocclusions can be corrected by orthodontic treatment by retraction of the upper and lower anterior teeth and myofunctional therapy for the perioral muscles.

Teeth are constantly exposed to pressure from perioral soft tissues such as the lips, cheeks, tongue, and superior pharyngeal constrictor, and the pressure balance influences the determination of the labiolingual position of the dentition [5].

Studies on the objective evaluation for the lip’s closure function have been dominated by two approaches: morphological and functional evaluation. A morphological evaluation includes analyses using cephalograms [6,7], 3D imaging [8], and MRI imaging [9], while a functional evaluation includes an electromyography of the perioral muscles [10] and a measurement of maximum lip closure force [11]. We focused on reports in the fields of sports science and physical therapy that state that the muscle tension is related to muscle stiffness [12].

There are many reports concerning the measurement of the elastic modulus for various parts of the human body [13,14,15,16,17,18,19,20,21,22,23,24,25,26,27,28,29,30,31,32,33,34,35,36,37]. For example, Pailler-Mattei et al. showed a dramatic decrease in Young’s modulus of skin with aging using an indenter approach [13]. They pointed out that a decrease in the density of collagen and elastic fibers is associated with the decrease in the elastic modulus. Their measurement method is basically a uniaxial compression, which gives the stress–strain curves for the skin tissues. Besides the mechanical measurements, Diridollou et al. developed a method to determine Young’s modulus for suctioned skin using an ultrasound [14]. The advantage of this method is that there is little variation in the measured values. In recent years, the measurement of the stiffness of human skin tissue using elastography has also been actively performed [15,16,17,18,19]. Also, there is a method using a composite of polymer film and a metal electrode, which has been successfully applied in practical use. When strain is applied to this device, the capacitance changes and parameters reflecting the elastic modulus of the muscle can be quantified. Although it is suitable for measuring at a large strain, the problem is that the measured values vary depending on the direction or the adhesion strength of the sensor attached to the muscle. Thus, a device and its methodology to determine Young’s modulus for soft tissues, such as perioral tissue easily and reproducibly, have not been established yet. As mentioned above, the conventional methods of evaluating the softness and tension of perioral tissues require large-size equipment, making it difficult for patients to accept the examination. For this reason, it has been extremely difficult to collect medical data through examinations at university hospitals. If the softness of the perioral tissues of patients could be evaluated quickly and easily at the chairside during examinations, a vast amount of data on various patients could be accumulated, and this field could be greatly advanced. Furthermore, if the device developed in this study can be miniaturized, it could be used like a wearable device that can measure the tension and activity of each region of the perioral muscles in real time [38,39].

In this study, we developed an apparatus that can measure the elastic modulus of perioral tissue using an elastic material made of polyurethane, as a model material of bi-ological tissues, for the final purpose of quantifying lip closure function. Polyurethane is a material that can reproduce a very wide range of softness and is widely used as a material similar to human skin in the field of medicine, including as a medical training model for suturing [40,41], facial plastic surgery [42], and chest tube insertion [43]. This apparatus is specially designed so that the stress can be dispersed to the displacement sensor for measuring the restoring force of soft materials with low elasticity.

## 2. Experimental Procedures

### 2.1. Synthesis of Polyurethane Elastomers

Polyurethane (PU) elastomers were used as a model material for perioral tissues. In this material, the elastic modulus can be controlled by either the number of cross-linking points or the network density. The number of cross-linking points and the network density can be controlled by the amount of cross-linking agent and the amount of plasticizer concentration, respectively. In this study, the plasticizer concentration varied while keeping the cross-linking density in order to vary the elasticity of polyurethane elastomers. The PU elastomers were synthesized by the prepolymer method. The prepolymer was synthesized by two kinds of polypropylene glycols (PPG), two-arm PPG with a molecular weight of *M*_w_ = 2000 (Fujifilm Wako Pure Chemical Co., Osaka, Japan) and three-arm PPG with a molecular weight of *M*_w_ = 3000 (Fujifilm Wako Pure Chemical Co., Osaka, Japan), and tolyrene diisocyanate (Fujifilm Wako Pure Chemical Co., Osaka, Japan) of a chain extender. For the PU elastomer, cross-linking points were created by the three-arm PPG. The prepolymer, linear polypropylene glycols, and plasticizer were mixed by a mechanical mixer (MAZERUSTAR KK-250S, Kurabo Industries Ltd., Osaka, Japan) for several minutes. The plasticizer is dioctyl phthalate (DOP, Fujifilm Wako Pure Chemical Co., Osaka, Japan). The weight concentration of DOP, which was defined as the ratio of DOP to the matrix, varied from 70 to 83 wt.% to control the elasticity of the PU elastomers. The molar ratio of -NCO to -OH groups in the prepolymer was constant at 2.01 (=[NCO]/[OH]), in order to be the final molar ratio of 1.0. After mixing, the mixed solution was poured in an aluminum mold and was cured on a hot stage at 100 °C for 30 min. The sample was in a disk shape with a diameter of 40 mm and a thickness of 20 mm.

### 2.2. Mechanical Measurements by a Compression Apparatus

The stress–strain curve was measured for the PU elastomers at room temperature using a uniaxial compression apparatus (EZ-Test EZ-SX, Shimadzu Co., Kyoto, Japan) with a load cell of 500 N. The measurements were carried out at a compression speed of 60 mm/min below a strain of approximately 0.16. The stress is apparent stress, which was calculated from *σ* = *F*/*S*; *F* is the load and *S* is the area of the cross-section of the PU elastomer without deformation. At low strains, the stress was proportional to the strain for all the elastomers with various plasticizer concentrations. Young’s modulus for the PU elastomers was determined from the initial slope of the stress–strain curve. The mean value and the standard error of Young’s modulus averaging over three measurements for one sample were presented in the graphs.

### 2.3. Mechanical Measurements by the Indentation Apparatus Developed

**Figure 1:**
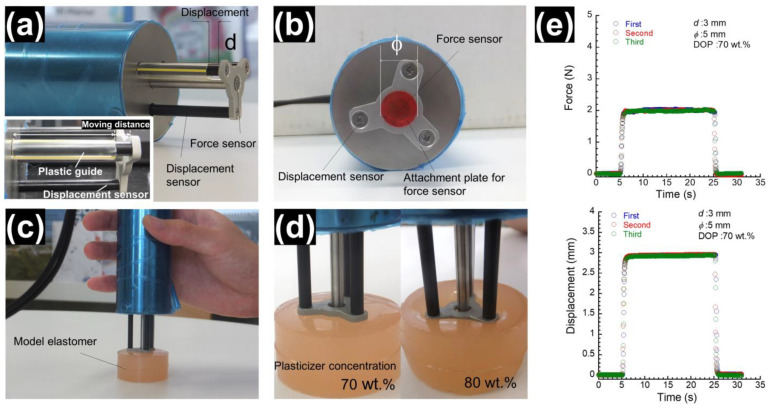
Photographs of the appearance of the (**a**) apparatus developed and for (**b**) force and displacement sensors. Views of (**c**) force measurement for a PU elastomer and of the (**d**) deformation of elastomers with Young’s moduli of 44 kPa (left) and 10 kPa (right). (**e**) Time response of force and displacement for PU elastomers with a plasticizer concentration of 70 wt.% when three indentation tests were carried out in approximately 3 min.

Photographs representing the new indentation apparatus developed in this study are shown in Figure 1. The indentation apparatus consists of a force sensor and a displacement sensor (Figure 1a). The load cell and the amplifier for the force measurement (Kyowa Electronic Instruments Co., Ltd., Kyoto, Japan) were used for measuring the force applied. The displacement was measured by a strain gage using a method of cantilevered spring, which is widely used for the mechanics of displacement detection. The displacement sensor also has a function of dispersing the restoring force received from the object. The spring constants for the force sensor and the displacement sensor were determined to be 4.8 × 10^3^ and 33 N/m, respectively, using the respective relationship between the force and displacement. The displacement can be adjusted by changing the length of a guide (1~3 mm) made of plastics (Figure 1a). The plastic guide restricts the maximum displacement of the displacement sensor, allowing the sensor to move to a set value. When the displacement sensor reaches a set value, force transfer occurs through the plastic guide. The diameter *ϕ* of the attachment plate mounted on the force sensor varied from 5 to 9 mm (Figure 1b). The force and displacement were measured by pressurizing the PU elastomers with the self-weight (=0.39 kg) of the developed apparatus, i.e., the measurement was performed without adding an additional force by hand (Figure 1c). First, the plastic guide was attached to the indentation apparatus; then, the apparatus was held with one hand and put on the PU elastomer or perioral tissues. While completing the measurement, it is important to set the indentation apparatus perpendicular to the surface of the elastomer. In the indentation method, the force sensor is often actively pressed into an object to be measured. In this case, the data scatters greatly depending on the experimental conditions, such as the contact condition and/or angle between the object and the apparatus, even though the indentation speed was controlled electrically. Needless to say, this significantly reduces the reproducibility of stress–strain curves. Figure 1d represents a photograph for PU elastomers with plasticizer concentrations of 70 and 80 wt.% when stress was applied by the self-weight of the apparatus. Load and displacement data were recorded by a computer using a software (TK-HS100P Ver. 1.20.1) via USB communication every 0.05 s. Figure 1e shows the time response of force and displacement for PU elastomers with a plasticizer concentration of 70 wt.% when three indentation tests were carried out in approximately 3 min. Similar curves of both force and displacement were observed at three runs, indicating that the test has high reproducibility even though indentation was completed by hand. The data also showed that the mechanical properties of the soft PUs were largely restored to their original state within this time. When the apparatus is applied to the skin, not only is the force sensor in contact with the skin, but so is the displacement sensor. In other words, the skin is pressurized in a certain plane. The values are almost the same, even though the perpendicularity of the indentation to the skin varied slightly with different examiners. The average value and error of the results of three different measurements are shown in the graph.

## 3. Results and Discussion

**Figure 2:**
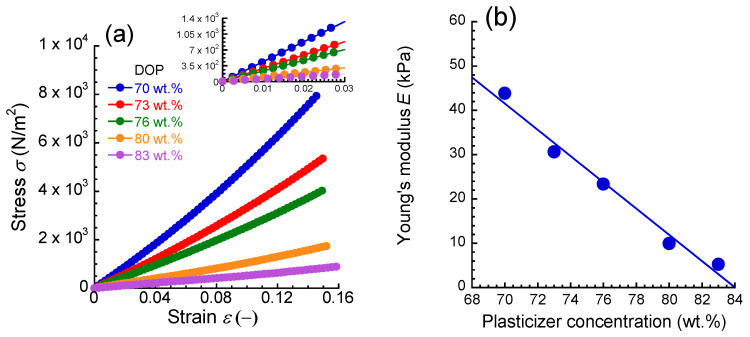
(**a**) Stress–strain curves and (**b**) the relationship between Young’s modulus and the plasticizer concentration for PU elastomers synthesized in this study.

Figure 2a shows the stress–strain curves for PU elastomers with various plasticizer concentrations measured by the uniaxial compression apparatus. The stress *σ* increased with the strain *ε*, and a region satisfying Hooke’s law (*σ* = *Eε*, *E*: Young’s modulus) was observed at strains below 0.03 for all the elastomers. Figure 2b exhibits the relationship between Young’s modulus and the plasticizer concentration for PU elastomers. Young’s modulus linearly decreased with the plasticizer concentration. PU elastomers with Young’s moduli from 5.3 kPa to 44 kPa can be obtained by controlling the plasticizer concentration. It is natural that the absolute value of Young’s modulus depends on the compression speed. However, the fact must remain that Young’s modulus decreases as the plasticizer concentration increases. Therefore, the absolute value of Young’s modulus of perioral tissue may be changed by changing the compression speed; however, its order of softness will not be changed. The deformation of elastomers with plasticizer concentrations of 70 and 80 wt.% can be seen in Figure 1d. This method developed here is similar to the principle of durometer used in the hardness of rubber (Shore hardness). Since the deformation is constrained around the measuring area, a theoretical equation is required to obtain the absolute value of Young’s modulus. In this study, we attempted to easily determine Young’s modulus from the magnitude of force by creating a calibration curve between the force, measured by the developed apparatus, and Young’s modulus, measured by a compression test. In general, a power-law relationship between the Shore hardness and Young’s modulus or the storage modulus (at several 10 Hz) can be observed in rubber materials, and it has been reported that these physical properties can be obtained from displacements measured by a durometer. For example, the hardness of rubber measured by a pneumatic tester can be scaled by Young’s modulus [44,45,46].

**Figure 3:**
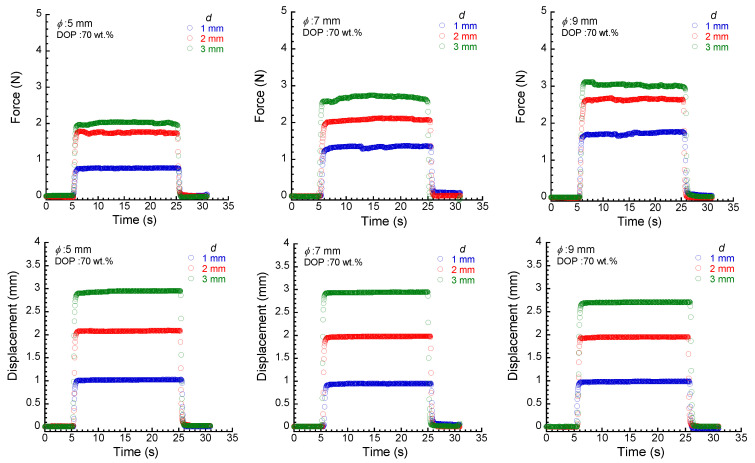
Time response of force (**tops**) and displacement (**bottoms**) for PU elastomers with a plasticizer concentration of 70 wt.% at various diameters of the force sensor, indicated as a function of the set values of displacement.

Figure 3 exhibits the time response of the force and the measured displacement for PU elastomers with a plasticizer concentration of 70 wt.% at various diameters of the force sensor, presented as a function of the set values of displacement. The force for all elastomers increased simultaneously with indenting the sensors in the elastomer, and it became almost constant within several seconds. Although the data fluctuated immediately after the displacement reached a set value, there was a stable region where the data were almost constant for approximately 10 s, which was used as the average force. When the indenter was removed from the elastomer, the force was completely recovered to the original value. A similar response to the force was observed for displacement. Thus, the measured displacement was equal to the set value when the diameter was small and the displacement was shallow. The measured displacement did not reach the set value when the diameter was large and the displacement was deep, e.g., *ϕ* = 9 mm and *d* = 3 mm.

**Figure 4:**
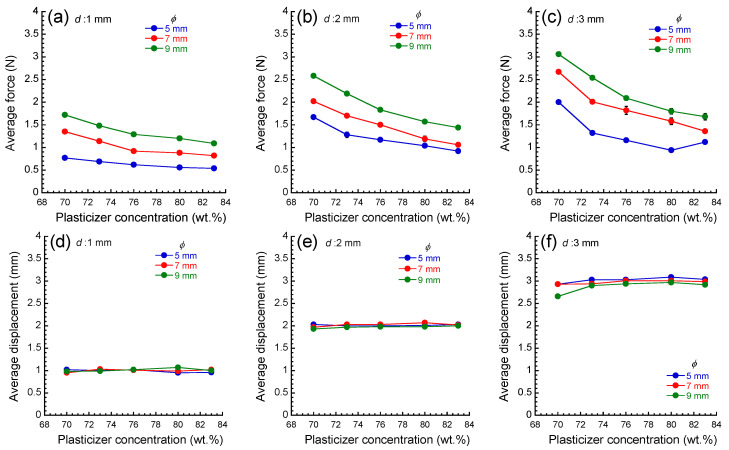
Average force (**a**–**c**) and average displacement (**d**–**f**) as a function of the diameter of the force sensor for PU elastomers with various plasticizer concentrations.

Figure 4 indicates the average force and average displacement as a function of the plasticizer concentration at various set values of the displacement as a function of the diameter of force sensor. The force decreased with the plasticizer concentration at all conditions, and it increased with increasing the diameter of the force sensor or increasing the displacement. The decrease in stress was significant at lower plasticizer concentrations and at deeper displacements. The decrease in stress was relatively small at higher plasticizer concentrations. The measured displacement was equal to the set value, indicating that the self-weight of the apparatus is enough to deform these elastomers. The average displacement for only an elastomer with a plasticizer concentration of 70 wt.% at *d* = 3 mm and *ϕ* = 9 mm did not reach the set value, which is due to the insufficient self-weight of the apparatus; this data was not dealt with in analyses hereafter. At a condition of *d* = 3 mm and *ϕ* = 5 mm, the force showed slightly high values at a plasticizer concentration of 83 wt.%. At this condition, the distance between the tip of the force sensor and the table under the elastomer was a few millimeters, that is, the elastomer is very thin. The relatively high value of the force might be an influence on the table, which does not show elastic deformation. In the measurement of the elastic modulus of human skin tissues, there should be an influence of bone under the skin tissues. The deformation of the bone is negligibly small compared to that of the skin tissues. Accordingly, at a deep indentation, the part that is deformed by stress is the skin tissue, and the skin tissue between the bone and the force sensor becomes very thin under the stress. This is similar to the indentation of PU elastomers sandwiched between the table and force sensor, as described above. For an accurate measurement of the indentation experiment for skin tissues, such as the slight and anomalous increase in the force, as seen in the force at a plasticizer concentration of 83 wt.%, it should be analyzed carefully.

**Figure 5:**
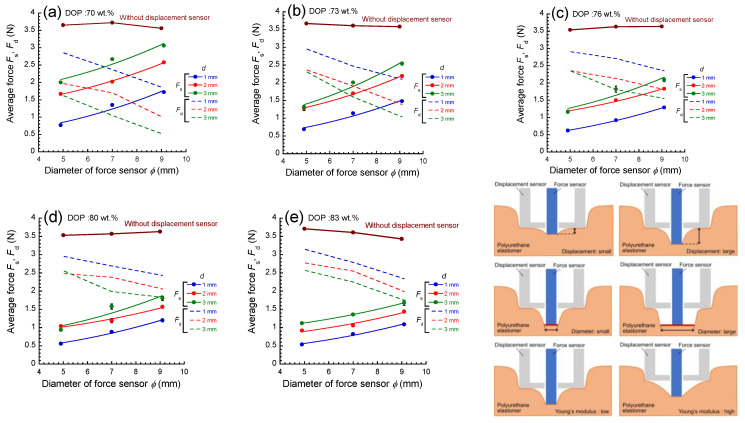
Relationship between the average force and the diameter of force sensor at various displacements for PU elastomers with various plasticizer concentrations. Schematic illustrations represent the deformation of elastomers around the force sensor when pressurized by the apparatus.

Figure 5 shows the relationship between the average force and the diameter of the force sensor at various displacements for PU elastomers with various plasticizer concentrations. The *F*_s_ was observed to increase with the displacement from 1 to 3 mm. This is because the restoring force by the elastomer increases as the distance between the top of the force sensor and the displacement sensor increases. The force increased with the diameter of the force sensor at all conditions, and it increased with displacement. The *F*_s_ was observed to increase with the diameter from 5 to 9 mm. This is because the restoring force, due to the elastomer, increases as the area of the deformed part increases. The *F*_s_ was observed to increase with Young’s modulus from 5.3 to 44 kPa. This is because the restoring force by the elastomer increases as Young’s modulus of the deformed part increases. The force applied to elastomers can be divided into two forces; one is a force received by the force sensor *F*_s_ and another is a force received by the displacement sensor *F*_d_,

(1) $$F_{s}+F_{d}=Wg$$

Here, the force *Wg* was calculated to be 3.8 N, which is a multiple of the mass of the apparatus, *W* (=0.39 kg), and the acceleration of gravity, *g* (=9.8 m/s^2^). The *F*_s_ should be proportional to the diameter of the force sensor as explained by the following equation:

(2) $$F_{s}=\frac{\pi \sigma_{s}}{4}\phi^{2}$$

Here, the *σ*_s_ and *ϕ* stand for the stress acting on the force sensor and the diameter of the force sensor. The *F*_s_ was well fitted by Equation (2), and the *σ*_s_ could be determined. On the other hand, the *F*_d_ was calculated from Equation (1), and it is indicated as dotted lines in Figure 5. The *F*_d_ decreased as the diameter of the force sensor at all conditions increased, and it increased with the decreasing displacement. At low plasticizer concentrations (high Young’s modulus), the *F*_s_ showed high values and the slope of the curves of *F*_s_ vs. *ϕ* was also high. At high plasticizer concentrations (low Young’s modulus), the *F*_s_ showed low values and the slope was also low.

The force measured without the displacement sensor is also shown in Figure 5. It should be noted that the force was almost constant, even though the diameter of the force sensor largely increased in spite of the plasticizer concentrations. Furthermore, the value of the force was approximately 3.6 N, which is 0.2 N smaller than that for the weight of the apparatus (=3.8 N). The small difference is due to the restoring force by the elastic deformation of the PU elastomers. These facts strongly indicate that the force measured without the displacement sensor hardly reflects the restoring force due to the deformation of PU elastomers occurring independently of the plasticizer concentration. This is rather similar to the mechanical response when the apparatus is placed on an object without any accompanying elastic deformation, e.g., a perfect solid. On the other hand, the force with displacement sensor showed a square dependence on *ϕ*, as shown in Equation (2), which is the mechanical response when the apparatus is placed on an object with an elastic deformation. Thus, it is clear that the mounting of the displacement sensor enables the measurement of the restoring force of PU elastomers. In this experiment, the diameter of the force sensor was set to be 9 mm at the maximum. The force received from the elastomers increased with the diameter of the sensor, which is appropriate for the measurement with high accuracy. However, the diameter should be less than 10 mm since there is a distribution of an elastic modulus in the plane for the measurements of perioral tissues.

**Figure 6:**
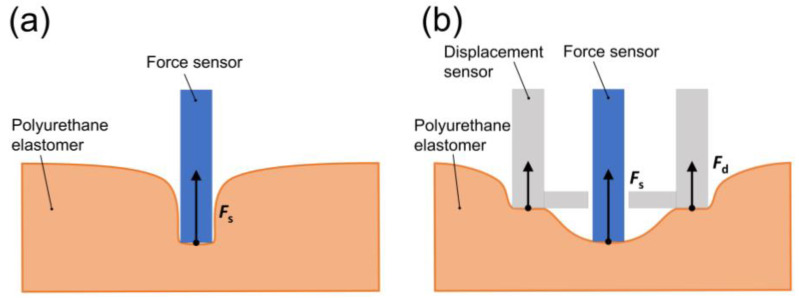
Schematic illustrations representing the deformation and mechanical response when a stress was applied by the indenter without (**a**) and with (**b**) a displacement sensor.

Figure 6 represents a schematic illustration representing the deformation and mechanical response of PU elastomers when the apparatus was placed on the elastomer. Without the displacement sensor, the force sensor deeply sinks into the elastomer. The force measured was almost independent of the diameter of the force sensor, as shown in Figure 5. This is not strange, although there is a part in the elastomer that is largely deformed by the indenter (Figure 6a). This can be understood as an effect of stress dispersion. In the presence of a displacement sensor, the force sensor sinks into the elastomer, similar to the case without the displacement sensor. However, the deformation of the elastomer around the force sensor is different. The elastomer within the circle of the displacement sensor is deformed by the force sensor (Figure 6b), which gives a general relationship between force and displacement. Actually, the force increased with the diameter of the force sensor and it decreased with the plasticizer concentration, as shown in Figure 5, which is a typical response of the restoring force by deformation for soft materials.

The general indenter test for human skin tissues is mechanically similar to the measurement in this study; however, most of them measure the force while increasing the displacement by indentation [13,20]. As mentioned above, the stress–strain curve often scatters greatly depending on the experimental conditions, even if the indentation speed is controlled electrically. This disadvantage makes it impossible to simply compare data obtained from different measurers and subjects. Although the mechanics are slightly complicated by mounting the displacement sensor, it acts to enhance the restoring force of the soft material due to deformation.

**Figure 7:**
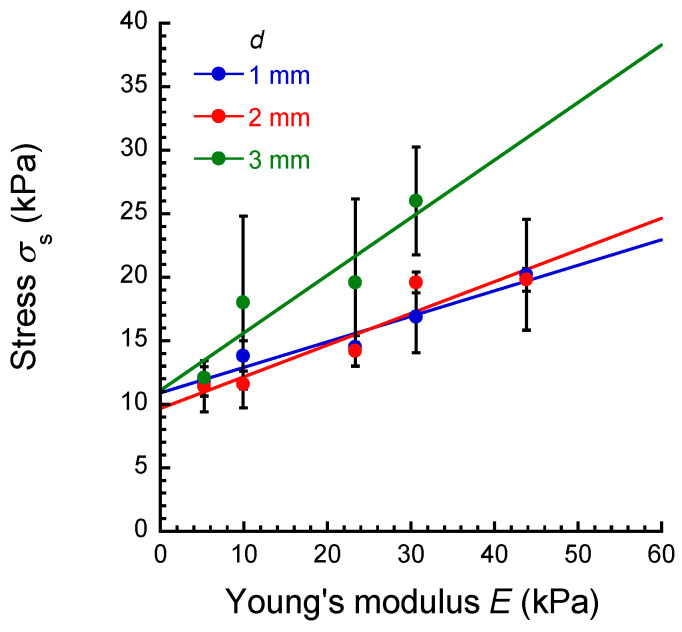
The relationship between the stress measured by the apparatus developed in this study and Young’s modulus determined by a uniaxial compression apparatus for PU elastomers with various plasticizer concentrations at various displacements.

Figure 7 shows the relationship between the stress measured by the apparatus developed in this study and Young’s modulus is determined by a uniaxial compression apparatus for PU elastomers with various plasticizer concentrations. Errors were obtained when the data of *σ*_s_ in Figure 5 were fitted using the least squares method according to Equation (2). The *σ*_s_ increased in proportion to Young’s modulus at all displacements, meaning that Young’s modulus for an object can be calculated from *σ*_s_ at any displacement studied here. However, the slope of the lines increased as the displacement increased, suggesting that Young’s modulus can be determined accurately at high displacements. Thus, the optimum displacement for measuring Young’s modulus for perioral tissues in clinical tests was determined to be 3 mm.

**Figure 8:**
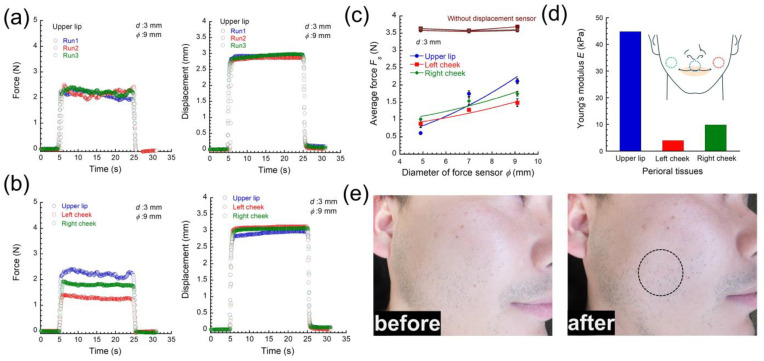
Time responses of (**a**) force and (**b**) displacement for perioral tissues measured at *ϕ* = 9 mm and *d* = 3 mm. (**c**) The relationship between the average force and the diameter of the force sensor for perioral tissues at a displacement of 3 mm. (**d**) Young’s modulus in vivo for perioral tissues. (**e**) The appearance of the skin surface before and after three indentation tests. The indenter was applied to the circle in (**e**).

Young’s modulus of perioral tissues for subjects who were healthy males in their 20s was measured using the apparatus developed in this study. The restoring force by the perioral tissues, lips, and cheek, of the subjects was measured. During the measurement, the subject was lying on their back, making a relaxed state for perioral tissues so that the back teeth were in contact and the mouth was not completely closed. The self-weight of the apparatus was applied to the tissue perpendicularly to its surface. In our clinical tests, the time interval between the measurements was kept at approximately 1 min. The interval is considered to be enough for the tissue to recover to its original state, since the force was recovered to the original value when removing the force typically within ~2 s. Figure 8a exhibits the time response of the force and the measured displacement for upper lip measured at *ϕ* = 9 mm and *d* = 3 mm. Three measurements were performed in approximately three minutes. The responses of both force and displacement for perioral tissues were similar to those of PU elastomers, as shown in Figure 3. This means that the similar procedures of measurement and analysis used in PU elastomers can be applied in perioral tissues. Figure 8b shows the time response of the force and the measured displacement for perioral tissues measured at the same condition mentioned above. The data indicated that the mechanical properties of the perioral tissues were restored to almost their original state within this time. Figure 8c shows the relationship between the average force and the diameter of the force sensor at a displacement of 3 mm for upper lip, left cheek, and right cheek. At all perioral tissues, the force increased with increasing the diameter of the force sensor and with increasing the displacement, as well as the results of indentation for the PU elastomers. Although a displacement of 3 mm was relatively deep, the actual displacement completely reached the set value also for the perioral tissues. It was also found that the *F*_s_ could be well fitted by Equation (2) with high values for the correlation coefficient, as shown in Figure 5. The force measured without the displacement sensor is also shown in Figure 8c. The force for perioral tissues was found to be almost constant, even though the diameter of the force sensor largely increased, which was similar to the result of PU elastomers. Using a similar manner as shown in Figure 5, the values of *σ*_s_ for these perioral tissues were determined to be 31, 13, and 16 kPa, respectively. Hence, the values of Young’s modulus were also evaluated to be 45, 4.0, and 9.9 kPa, respectively. According to a sensory evaluation of tactile sense, the upper lip was stiffer than the right and left cheeks, which was consistent with the experimental values. The result where Young’s modulus of the upper lip was several times higher than that of the cheeks can be attributed to the influence of hard teeth behind the lip. The limitation of the measurement with this apparatus is that when the object to be measured is extremely soft, the influence of the substrate under the object will be more prominent. This problem is a phenomenon that is generally found in indentation tests of soft materials, which cannot be avoided in principle and is a limitation of this apparatus. We are currently investigating what part of the measurements in clinical tests where this problem could lead to inaccuracies in Young’s modulus. On the other hand, there was no difference in softness between the left and right cheeks according to the tactile sensory evaluation, although the experimental values showed a difference of 5.9 kPa. This apparatus enables the measurement of small differences in softness that cannot be sensed by tactile sense by fingers. The reason for the difference in Young’s modulus between the left and right cheek tissues is still unknown. We recognize empirically that there is an asymmetry in muscle tension on the left and right sides of the face. Actually, it has long been noted that the temporalis muscle and the masseter muscle, one of the perioral muscles, are asymmetrical, and that the masseter muscles tend to be more variable than the other muscles [47]. However, numerical values have not been clarified yet due to underdeveloped methodologies for measurement and a lack of clinical data. Using the apparatus developed in this study, we aim to measure Young’s modulus of each muscle around the lips to establish a criterion for cosmetics, such as apparent symmetry, and for eating functions, such as biting. As mentioned in the Section 1, there are several methods to measure the elastic modulus for skin tissues such as indentation measurement [13,20,21], suction techniques [14,22,23,24,25,26], torsion systems [27,28,29,30,31], traction [32], extensometry [33,34,35], and elastic wave propagation [36,37]. A study using an indentation apparatus by the push-in method reports that Young’s modulus for the left cheek near the lips and for the center of the left jaw is distributed in a range of 2.653–4.437 kPa, and for the center of the cheek, the left zygomatic region, and the left cheek near the lips is distributed in a range of 1.649–3.395 kPa [21]. These values of Young’s modulus were close to the lower values measured with our apparatus. In the literature on larger muscles, Young’s moduli for medial forearm was identified as 5.1–12.3 kPa by an indentation test [13], and 129 ± 88 kPa by a suction test using an echorheometer [14]. In the future, we plan to measure various tissues of anatomy in clinical tests to validate Young’s moduli, as we presented in this study. Here, the effect of surface curvature on the measured value should be discussed. At this stage, we believe that the elastic modulus can be measured even if the object to be measured has a curved surface. However, the possibility that curved surfaces may affect the experimental results cannot be completely eliminated. Extensive experiments on samples with a curved surface are needed to prove this idea. The reasons why measurements can be made even on curved surfaces are as follows. Basically, the object being measured with this apparatus is soft, like human skin tissue. When the apparatus is placed on the skin, the displacement and force sensors sink into the skin due to the self-weight of the apparatus. Due to this, the area where the force sensor contacts the surface will be flat, even if the surface is rough. In other words, the restoring force is measured in this apparatus when a limited area near the force sensor is deformed. Figure 8e shows the appearance of the skin surface before and after three indentation tests using an indenter with a diameter of 9 mm at a displacement of 3 mm. In clinical tests, there was no change in the appearance of the skin surface after the indentation, meaning that the skin was not damaged by the indentation. The apparatus developed here has the following advantages in clinical tests. In general, push-in devices are large and heavy; however, this apparatus is compact and portable, as shown in Figure 1. Also, the displacement sensor has roles in not only enhancing the restoring force but also in dispersing the force applied to the skin, resulting in a painless measurement. We conducted a clinical test on one patient because we wanted to show in this paper that the newly developed apparatus could actually be used in a clinical test. The results of a clinical test on a statistically sufficient number of patients will be presented in a subsequent paper.

## 4. Conclusions

We have fabricated an apparatus to evaluate Young’s modulus for perioral tissues accurately and easily in clinical tests. It was specially designed so that the stress can be dispersed to the displacement sensor for measuring the restoring force of perioral tissues with low elasticity accurately. Mechanical measurements for polyurethane elastomers with various elastic moduli, which is a model material of perioral tissues, were carried out using the apparatus, and a methodology was developed to convert the measured stresses to Young’s modulus. An optimized condition was derived, so that the displacement was 3 mm. In a clinical test, a difference in Young’s modulus between the left and right cheeks could be detected by the apparatus. This method has the advantage of high reproducibility and being inspector independent, since the measurement is performed using the self-weight of the apparatus. We believe that the apparatus developed and the mechanical findings obtained are powerful tools for evaluating the elasticity of perioral tissues. The present apparatus is possibly very helpful to predict the difference in the response of perioral tissue to orthodontic tooth movement in individual cases. Using the apparatus developed in this study, we aim to measure Young’s modulus of each muscle around the lips to establish a criterion for cosmetics, such as apparent symmetry, and for eating functions, such as biting.

## Data Availability

Data are contained within the article.

## References

[1] Holden A.C.L. (2018). Cosmetic dentistry: A socioethical evaluation. Bioethics.

[2] Shimomura T., Ioi H., Nakata S., Counts A.L. (2011). Evaluation of well-balanced lip position by Japanese orthodontic patients. Am. J. Orthod. Dentofac. Orthop..

[3] Al-Namankany A., Alhubaishi A. (2018). Effects of cleft lip and palate on children’s psychological health: A systematic review. J. Taibah Univ. Med. Sci..

[4] Burstone C.J. (1967). Lip posture and its significance in treatment planning. Am. J. Orthod..

[5] Howland J.P., Brodie A.G. (1966). Pressures exerted by the buccinator muscle. Angle Orthod..

[6] Downs W.B. (1948). Variations in facial relationships: Their significance in treatment and prognosis. Am. J. Orthod..

[7] Holly Broadbent B. (1931). A new X-ray technique and its application to orthodontia. Angle Oathod..

[8] Revilla-León M., Zeitler J.M., Blanco-Fernández D., Kois J.C., Att W. (2022). Tracking and recording the lip dynamics for the integration of a dynamic virtual patient: A novel dental technique. J. Prosthodont..

[9] Basford J.R., Jenkyn T.R., An K.N., Ehman R.L., Heers G., Kaufman K.R. (2002). Evaluation of Healthy and Diseased Muscle with Magnetic Resonance Elastography. Arch. Phys. Med. Rehabil..

[10] Moyers R.E. (1949). Temporomandibular muscle contraction patterns in Angle Class II, Division 1 malocclusions: An electromyographic analysis. Am. J. Orthod..

[11] Hijiya K., Masuda Y., Miyamoto T., Shimono R., Kato T., Kageyama T., Yamada K. (2021). Age-related differences in maximum voluntary lip-closing force and ability to control lip-closing force. J. Oral Biosci..

[12] Igawa K., Kashima K., Maeda S., Shiba R. (2003). Measurement of muscle hardness using a hardness meter: Application to the masseter and temporal muscles and reproducibility of measurement. Cranio.

[13] Pailler-Mattei C., Debret R., Vargiolu R., Sommer P., Zahouani H. (2013). In vivo skin biophysical behaviour and surface topography as a function of ageing. J. Mech. Behav. Biomed. Mater..

[14] Diridollou S., Patat F., Gens F., Vaillant L., Black D., Lagarde J.M., Gall Y., Berson M. (2000). In vivo model of the mechanical properties of the human skin under suction. Ski. Res. Technol..

[15] Lung C.-W., Wu F.-L., Zhang K., Liau B.-Y., Townsend R., Jan Y.-K. (2020). Using Elastographic Ultrasound to Assess Plantar Tissue Stiffness after Walking at Different Speeds and Durations. Appl. Sci..

[16] Billy J., Bensamoun S.F., Mercier J., Durand S. (2024). Applications of ultrasound elastography to hand and upper limb disorders. Hand Surg. Rehabil..

[17] Bedewi M.A., Elsifey A.A., Alfaifi T., Saleh A.K., Swify S.M., Sandougah K.J. (2021). Shearwave elastography of the Sartorius muscle. Medicine.

[18] Kronlage C., Grimm A., Romano A., Stahl J.-H., Martin P., Winter N., Marquetand J. (2021). Muscle Ultrasound Shear Wave Elastography as a Non-Invasive Biomarker in Myotonia. Diagnostics.

[19] Olchowy C., Więckiewicz M., Sconfienza L.M., Łasecki M., Seweryn P., Smardz J., Hnitecka S., Dominiak M., Olchowy A. (2020). Potential of Using Shear Wave Elastography in the Clinical Evaluation and Monitoring of Changes in Masseter Muscle Stiffness. Pain Res. Manag..

[20] Pailler-Mattei C., Bec S., Zahouani H. (2008). In vivo measurements of the elastic mechanical properties of human skin by indentation tests. Ski. Res. Technol..

[21] Wei H., Liu X., Dai A., Li L., Li C., Wang S., Wang Z. (2021). In Vivo Measurement of the Mechanical Properties of Facial Soft Tissue Using a Bi-Layer Material Model. Int. J. Appl. Mech..

[22] Hendriks F., Brokken D., Van Eemeren J., Oomens C., Baaijens F., Horsten J. (2003). A numerical-experimental method to char-acterize the non linear mechanical behaviour of human skin. Skin. Res. Technol..

[23] Grahame R., Holt P. (1969). The Influence of Ageing on the in vivo Elasticity of Human Skin. Gerontology.

[24] Grahame R. (1969). Elasticity of human skin in vivo. A study of the physical properties of the skin in rheumatoid arthritis and the effect of corticosteroids. Ann. Phys. Med..

[25] Alexander H., Cook T. (2006). Variations with age in the mechanical properties of human skin in vivo. J. Tissue Viability.

[26] Barel A.O., Lambrecht R., Clarys P. (1998). Mechanical function of the skin: State of the art. Curr. Probl. Dermatol..

[27] Sanders R. (1973). Torsional elasticity of human skin in vivo. Pflug. Arch. Eur. J. Physiol..

[28] Berardesca E., De Rigal J., Leveque J., Maibach H. (1991). In vivo biophysical characterisation of skin physiological differences in races. Dermatologica.

[29] Agache P.G., Monneur C., Leveque J.L., De Rigal J. (1980). Mechanical properties and Young’s modulus of human skin in vivo. Arch. Dermatol. Res..

[30] De Rigal J., Leveque J.L. (1985). In vivo measurement of the stratum corneum elasticity. Bioeng. Skin..

[31] Escoffier C., Pharm M., De Rigal J., Rochefort A., Pharm M., Vasselet R., Leveque J.L., Agache P.G. (1989). Age-related mechanical properties of human skin: An in vivo study. J. Investig. Dermatol..

[32] Sugihara T., Ohura T., Homma K., Igawa H. (1991). The extensibility in human skin: Variation according to age and site. Br. J. Plast. Surg..

[33] Alexander H., Cook T. (1977). Accounting for natural tension in the mechanical testing of human skin. J. Investig. Dermatol..

[34] Özyazgan I., Liman N., Dursun N., Güneş I. (2002). The effects of ovariectomy on the mechanical properties of skin in rats. Maturitas.

[35] Del Prete Z., Antoniucci S., Hoffman A., Grigg P. (2004). Viscoelastic properties of skin in Mov-13 and Tsk mince. J. Biomech..

[36] Moran C., Bush N., Bamber J. (1995). Ultrasonic propagation properties of excised human skin. Ultrasound Med. Biol..

[37] Pan L., Zan L., Foster F. (1998). Ultrasonic and viscoelastic properties of skin under transverse mechanical stress in vitro. Ultrasound Med. Biol..

[38] Wang Y.H., Tang T.Y., Xu Y., Bai Y.Z., Yin L., Li G., Zhang H.M., Liu H.C., Huang Y.A. (2021). All-weather, natural silent speech recognition via machine-learning-assisted tattoo-like electronics: A Technical Note. npj Flex. Electron..

[39] Kohestanian M., Hasany M., Shabankareh A.N.T., Mehrali M. (2023). Fabricating of a custom 3D-printed setup for evaluating gel-based strain sensors. Alex. Eng. J..

[40] Antonopoulos I., Tzortzis A.S., Pechlivanidou E., Troupis T. (2023). A Simplified Three-Layered Suturing Training Pad for Undergraduate Medical Students: A Technical Note. Cureus J. Med. Sci..

[41] Nakamura K., Ogasawara M., Matsumoto K., Andoh H., Jikei M. (2023). Functional Expression of Low Puncture Resistance and Physical Property Evaluation of a Suture Training Model Made of Polyurethanes. J. Fiber Sci. Technol..

[42] Tripathy S., Mohapatra D.P., Thiruvoth F.M., Sharma R.K., Reddy L., Thomas N. (2022). An Innovative Skin Simulation Model to Augment Competency-Based Training in Facial Plastic Surgery. Indian J. Plast. Surg..

[43] Crawford S.B., Huque Y.I., Austin D.E., Monks S.M. (2019). Development and Review of the Chest Tube High-Feedback Educational Simulation Trainer (CHEST). Simul. Health J. Soc. Simul. Health.

[44] Kucherskii A., Kaporovskii B. (1995). Problems in determining hardness of rigid rubbers. Polym. Test..

[45] Kucherskii A., Kaporovskii B. (1997). A promising method for measuring hardness of rubbers. Polym. Test..

[46] Bouaziz R., Truffault L., Borisov R., Ovalle C., Laiarinandrasana L., Miquelard-Garnier G., Fayolle B. (2020). Elastic Properties of Polychloroprene Rubbers in Tension and Compression during Ageing. Polymers.

[47] Spronsen P.H.V., Weijs W.A., Valk J., Prahl-Andersen B., Ginkel F.C.V. (1991). Relationships between jaw muscle cross-sections and craniofacial morphology in normal adults, studied with magnetic resonance imaging. Eur. J. Orthod..

